# Systematic reviews on tranexamic acid in shoulder arthroplasty frequently exhibit reporting bias

**DOI:** 10.1016/j.xrrt.2026.100696

**Published:** 2026-02-16

**Authors:** Jason Fink, Dogerno J. Norceide, Arjan Kahlon, Kenneth T. Nguyen, Kevin A. Wu, Eric Mai, Avanish Yendluri, Erin L. Brown, John D. Kelly, Xinning Li, Robert L. Parisien

**Affiliations:** a Philadelphia College of Osteopathic Medicine, Philadelphia, PA, USA; b Drexel College of Medicine, Philadelphia, PA, USA; c Department of Orthopaedics, University of Pennsylvania, Philadelphia, PA, USA; d Tulane University School of Medicine, New Orleans, LA, USA; e Department of Orthopaedics, Icahn School of Medicine at Mount Sinai, New York, NY, USA; f Department of Orthopaedics, Boston University, Boston, MA, USA

**Keywords:** Arthroplasty/replacement/shoulder, Blood loss/surgical, Classification, Tranexamic acid, Clinical decision-making

## Abstract

**Background:**

Tranexamic acid (TXA) is increasingly used in total shoulder arthroplasty to minimize perioperative blood loss; however, the quality and objectivity of evidence supporting its use remain uncertain. Systematic reviews and meta-analyses are critical for informing clinical practice, but their conclusions can be affected by reporting bias—particularly spin, which may distort study findings and mislead clinical decision-making. Despite the rising interest in TXA for total shoulder arthroplasty, the extent to which spin is present in this body of literature has not been evaluated. Therefore, the purpose of this study was to analyze reporting bias in the form of spin present in systematic reviews and meta-analyses related to TXA use in shoulder arthroplasty.

**Methods:**

Following the Preferred Reporting Items for Systematic Reviews and Meta-Analyses guidelines, systematic reviews were collected from PubMed, Scopus, and Embase using the search “Shoulder Arthroplasty” AND “Tranexamic Acid” OR “TXA” AND “systematic review” OR “meta-analysis” in March of 2025. A MeaSurement Tool to Assess systematic Reviews (AMSTAR 2) was used to evaluate the quality of the studies. Associations between these factors and spin presence or type were determined using statistical tests, including *t*-tests, one-way analysis of variance, Fisher tests, and Spearman's rank coefficients.

**Results:**

The initial database search identified 72 studies, of which 31 duplicates were removed. Twenty-one additional studies were removed after title and abstract screening, and 5 more were removed during full-text screening because they did not meet the inclusion criteria. Spin was present in 12 out of the 15 remaining study abstracts (80.0%), all of which were determined to contain misleading reporting, and 10 (66.7%) of which were also found to contain misleading interpretation. Furthermore, 12 of the 15 reviews (80.0%) received a critically low AMSTAR 2 confidence rating.

**Conclusion:**

The majority of systematic reviews on TXA use in shoulder arthroplasty received critically low AMSTAR 2 ratings, reflecting the poor quality of evidence in this area. This analysis highlights the widespread occurrence of spin and low-quality evidence in TXA use in shoulder arthroplasty reviews, underscoring the importance of critical evaluation and improved research quality.

Total shoulder arthroplasty (TSA) is a commonly performed surgical procedure for the treatment of glenohumeral arthritis and other degenerative conditions of the shoulder. In recent years, the use of tranexamic acid (TXA), an antifibrinolytic agent, has gained popularity in orthopedic surgery for its potential to reduce perioperative blood loss and the need for transfusions.^2^ While TXA has demonstrated efficacy in lower extremity arthroplasty, its benefits in TSA remain an area of ongoing investigation.

As clinical evidence supporting the use of TXA in TSA grows, it becomes increasingly important to assess the integrity and objectivity of how these data are reported in the literature. One concern is the presence of spin, which refers to a specific way of reporting results—either intentionally or unintentionally—that may exaggerate benefits or understate potential harms of an intervention.^7^^,^^22^ Spin can distort the perceived efficacy or safety of a treatment and lead to misinformed clinical decision-making.

Spin in scientific literature can take several forms, including misleading interpretations of results, selective reporting, and unwarranted extrapolations.^7^^,^^20^ Prior studies have demonstrated that spin is frequently found in study abstracts, which clinicians often rely on when forming first impressions about a treatment's value. When present, spin can influence the perceived validity and clinical applicability of findings, especially in systematic reviews and meta-analyses, which are often cited as high levels of evidence.

Given the growing interest in TXA use for TSA and the critical role of high-quality evidence in guiding clinical practice, it is essential to evaluate the transparency and accuracy of how such data are communicated. The purpose of this study was to assess the presence and types of spin in abstracts of systematic reviews and meta-analyses evaluating TXA in TSA. While this study examines the quality of reporting its evidence, it does not revisit the well-established safety and efficacy of TXA use. We hypothesized that spin would be present in a significant proportion of abstracts and that higher levels of spin would be associated with lower methodological quality of the studies.

## Methods

This study was conducted according to the Preferred Reporting Items for Systematic Reviews and Meta-Analyses (PRISMA) guidelines using a predetermined protocol. Three authors (D.J.N., A.K., and J.F.) conducted a search of the PubMed, Scopus, and Embase databases using “Shoulder Arthroplasty” AND “Tranexamic Acid” OR “TXA” AND “systematic review” OR “meta-analysis” in March 2025. The search results were aggregated and deduplicated into Covidence. Two authors (D.J.N. and A.K.) independently screened the studies for eligibility, with a third author (J.F.) resolving any discrepancies.

Systematic reviews and meta-analyses related to TXA use in shoulder arthroplasty published in an English peer-reviewed journal were eligible for inclusion. Databases were queried from inception to March 2025. Studies were excluded if they were not peer-reviewed, not published in English, not systematic reviews and/or meta-analyses, retracted or withdrawn, included nonhuman or cadaver subjects, unrelated to shoulder arthroplasty and TXA use, published without an abstract, or did not have full text available.

Three authors (D.J.N., A.K., and J.F.) were trained using previously graded examples to identify study designs and characteristics and in the definition and classification of the most common types of spin proposed by Yavchitz et al as summarized in Table I.^22^ The authors also learned to assess study quality using A Measurement Tool to Assess Systematic Reviews 2 (AMSTAR 2).^5^ The adoption of AMSTAR 2 for assessing study quality is supported by its impressive inter-rater reliability and high construct validity.^17^ The authors collaborated using previous peer-reviewed articles that had applied the Yavchitz et al framework and AMSTAR 2 tool.

**Table I:** Description of types of spin assessed.

Category	Type	Description
Misleading interpretation	1	The conclusion formulates recommendations for clinical practice not supported by the findings
	2	The title claims or suggests a beneficial effect of the experimental intervention not supported by the findings
	4	The conclusion claims safety based on nonstatistically significant results with a wide confidence interval
	9	The conclusion claims the beneficial effect of the experimental treatment despite reporting bias
	12	The conclusion claims equivalence or comparable effectiveness for nonstatistically significant results with a wide confidence interval
Misleading reporting	3	Selective reporting of or overemphasis on efficacy outcomes or analysis favoring the beneficial effect of the experimental intervention
	5	The conclusion claims the beneficial effect of the experimental treatment despite a high risk of bias in primary studies
	6	Selective reporting of or overemphasis on harm outcomes or analysis favoring the safety of the experimental intervention
	10	Authors hide or do not present any conflict of interest
	11	The conclusion focuses selectively on statistically significant efficacy outcome
	13	Failure to specify the direction of the effect when it favors the control intervention
	14	Failure to report a wide confidence interval of estimates
Inappropriate extrapolation	7	The conclusion extrapolates the review findings to a different intervention (eg, claiming efficacy of one specific intervention although the review covered a class of several interventions)
	8	The conclusion extrapolates the review's findings from a surrogate marker or a specific outcome to the global improvement of the disease
	15	The conclusion extrapolates the review's findings to a different population or setting

*PROSPERO*, The International Prospective Register of Systematic Reviews.

Using AMSTAR 2, a 16-question critical appraisal tool, each study was graded on its methodological quality and assigned a confidence rating. This tool evaluates an author's incorporation of a predetermined study protocol, funding source, conflicts of interest, and an author’s overall ability to adequately characterize the findings of studies included in the review. The full texts of the included studies were used to assess study quality per the AMSTAR 2 checklist, which yielded a numeric score between 0 and 16. This score reflects the extent to which the studies meet quality standards, with the numerical value representing the number of AMSTAR 2 checklist elements found in the study. In addition to the numerical score, the AMSTAR 2 critical appraisal tool distinguishes between deficiencies in critical and noncritical domains. In doing so, the AMSTAR 2 assessment identifies critical flaws in systematic reviews and meta-analyses by assigning studies critically low, low, moderate, and high confidence ratings.^11^^,^^22^

Two authors (D.J.N. and A.K.) extracted data independently. In the case of disagreement, resolution was achieved after discussion between the 2 authors or input from a third author (J.F.). Study characteristics that were extracted included title, authors, publication year, study design, journal, funding source, level of evidence, reported adherence to PRISMA guidelines, preregistration status, and outcome measures. If not stated within the study, the level of evidence was determined using the American Academy of Orthopaedic Surgeons recommendations.

Each abstract was assessed for the 15 most common types of spin (Table I).^22^ The full texts of the included systematic reviews were used to assess study quality per the AMSTAR 2 checklist, which yielded a numeric score between 0 and 16. The AMSTAR 2 confidence ratings were also extracted. Additionally, the impact factor was recorded for the journals in which the included systematic reviews and meta-analyses were published. This metric, which gauges the significance of a journal by totaling the number of citations its selected articles receive over a recent period (typically the past few years), serves as a valuable tool for comparing journals within a specific subject category. A higher impact factor indicates a more esteemed journal. Impact factors were recorded using each journal's value for the year of publication.

### Data analysis

Descriptive statistics were used to characterize the frequency of spin occurring in the included studies. Study characteristics including study type, Level of Evidence, funding source, PRISMA adherence, The International Prospective Register of Systematic Reviews (PROSPERO) registration, impact factor, and AMSTAR 2 confidence rating were analyzed. Their association with the presence of spin, as well as the number of spin types present, was determined using Fisher exact test, Kruskal-Wallis test, Mann-Whitney U test, point-biserial correlation, logistic regression, and Spearman's rank correlation coefficients. All statistical analyses were performed using Python 3.11 with the SciPy library. Nonparametric tests were selected due to small sample size, non-normal distributions, and ordinal data characteristics. A *P* value <.05 was considered statistically significant.

## Results

### Overview of article screening

**Figure 1:**
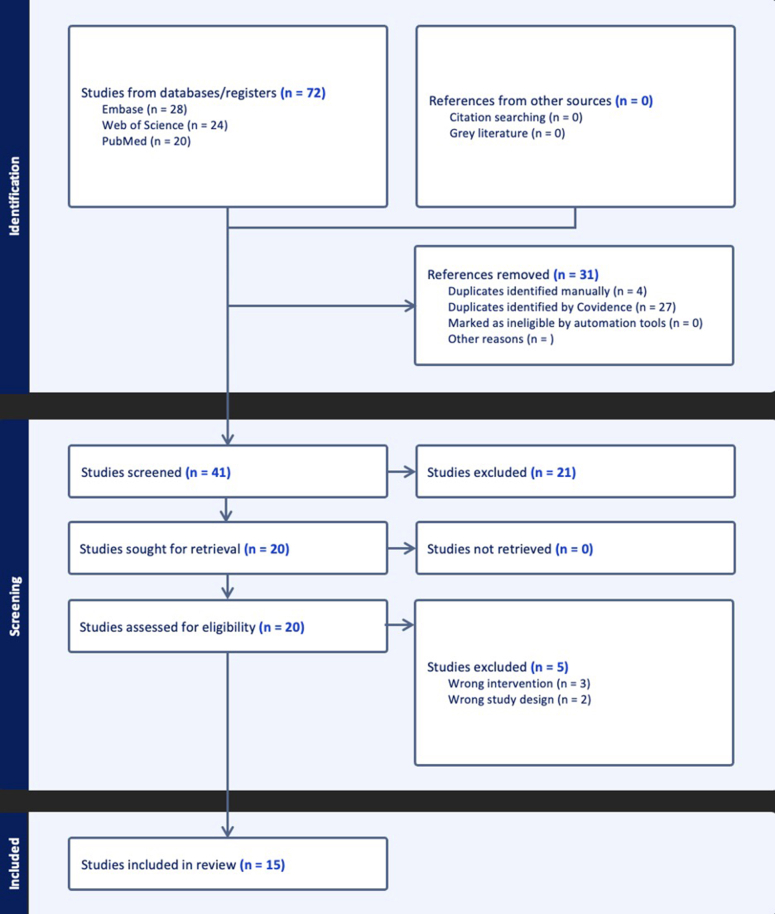
Preferred Reporting Items for Systematic Reviews and Meta-Analysis (PRISMA) flow diagram.

The initial database search identified 72 studies, of which 31 duplicates were removed. An additional 21 studies were removed after title and abstract screening because they did not meet the inclusion criteria. The remaining 20 studies were assessed for eligibility. Among the 20 studies, 5 (25.0%) were excluded—2 (40.0%) for using an incorrect study design and 3 (60.0%) for investigating an unrelated intervention. The 15 (75.0%) remaining systematic reviews and meta-analyses, which were published in 10 different journals with dates of publication ranging from 2017 to 2024, were included for analysis (Fig. 1).^1^^,^^3^, ^4^, ^5^, ^6^^,^^8^, ^9^, ^10^^,^^12^^,^^15^^,^^16^^,^^18^^,^^19^^,^^23^^,^^24^

### Indicators of quality analysis

**Figure 2:**
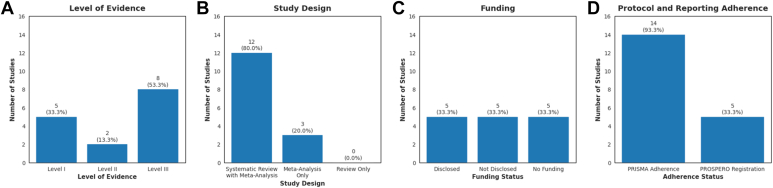
Distribution of quality indicators among the reviewed studies. (**A**) Level of evidence reported. (**B**) Study design classification. (**C**) Funding disclosure status. (**D**) Adherence to PRISMA guideline compliance and PROSPERO registration. Percentages reflect the proportion of studies within each category *PRISMA*, Preferred Reporting Items for Systematic Reviews and Meta-Analysis; *PROSPERO*, The International Prospective Register of Systematic Reviews.

Although Level of Evidence reflects trial design and not the reporting of trial results, it remained part of the analysis, as it provided a general idea of the included studies' architecture. Most of the included studies had a Level of Evidence III (8/15, 53.3%). There were 5 studies with a Level of Evidence of I (5/15, 33.3%) and 2 studies with a Level of Evidence of II (2/15, 13.3%) (Fig. 2*A*). Twelve studies (80.0%) included a systematic review with meta-analysis, while 3 studies (20.0%) were systematic reviews without meta-analyses (Fig. 2*B*). There were no systematic reviews without a meta-analysis. Five studies (33.3%) did not disclose funding, while 5 (33.3%) disclosed at least 1 external source of funding, and 5 (33.3%) received no funding (Fig. 2*C*). Five studies (33.3%) submitted their protocols to the PROSPERO public register of systematic reviews, and almost all studies reported PRISMA adherence (14/15, 93.3%) (Fig. 2*D*). Ten different journals were represented among these systematic reviews, for which the mean impact factor was 2.37 ± 1.44 (range: 0.31-5.3) (Table II).

**Table II:** Study characteristics of included systematic reviews and meta-analyses.

Author	Year published	Journal	Impact factor	PRISMA adherence	PROSPERO pre-registration
He et al^16^	2017	*The Journal of Medicine*	1.4	Y	N
Hartland et al^15^	2021	*The American Journal of Sports Medicine*	4.2	Y	Y
Kirsch et al^17^	2017	*The Journal of Bone and Joint Surgery*	5.3	Y	N
Tan et al^22^	2024	*Journal of Shoulder and Elbow Surgery*	2.8	Y	Y
Fan et al^14^	2021	*Journal of Shoulder and Elbow Surgery*	2.8	Y	Y
Rojas et al^20^	2020	*Journal Shoulder and Elbow Surgery*	1.5	Y	Y
Donovan et al^13^	2021	*Journal of Orthopaedics*	1.5	Y	Y
Sun et al^21^	2017	*Medicine*	1.8	Y	N
Pecold et al^19^	2021	*Journal of Clinical Medicine*	4.96	Y	N
Zhong et al^24^	2024	*BMC Musculoskeletal Disorders*	2.2	Y	N
Deng et al^12^	2023	*World Journal of Clinical Cases*	1.0	Y	N
Box et al^11^	2018	*Journal Shoulder and Elbow Surgery International*	0.31	Y	N
Kuo et al^12^	2018	*BMC Musculoskeletal Disorders*	2.2	Y	N
Yu et al^23^	2017	*Medicine*	2.03	N	N
Berk et al^10^	2024	*Journal Shoulder and Elbow Surgery*	1.5	Y	N

*PRISMA*, Preferred Reporting Items for Systematic Reviews and Meta-Analysis.

Studies with “Y” for PRISMA adherence indicates the study met the PRISMA criteria while an “N” indicates the study did not meet the criteria. Likewise, studies with “Y” for PROSPERO pre-registration indicates the systematic review protocols were pre-registered prior to beginning the study while an “N” indicates the systematic review was not pre-registered prior to beginning the study.

### AMSTAR 2 findings

Based on the AMSTAR 2 assessment, only 3 studies (20.0%) received a high confidence rating due to a lack of critical flaws. Twelve studies (80.0%) received a critically low confidence rating due to the presence of more than one critical flaw. No studies fell into the moderate confidence rating category, which required the presence of zero critical flaws, or the low confidence rating category, which required the presence of exactly one critical flaw. Any discrepancy in the AMSTAR 2 assessment between the authors was resolved with a discussion regarding semantics. The median AMSTAR 2 score was 11 (range: 6-16, mean = 11.47 ± 3.23) (Table III).

**Table III:** AMSTAR 2 assessment of reviewed studies.

AMSTAR 2 question (N = 15) (∗ = critical domain)	Yes	%
1. Did the research questions and inclusion criteria for the review include the elements of PICO?	15	100.0%
2. Did the report of the review contain an explicit statement that the review methods were established prior to the conduct of the review and did the report justify any significant deviations from the protocol?∗	4	26.7%
3. Did the review authors explain their selection of the study designs for inclusion in the review?	12	80.0%
4. Did the review authors use a comprehensive literature search strategy?∗	13	86.7%
5. Did the review authors perform study selection in duplicate?	12	80.0%
6. Did the review authors perform data extraction in duplicate?	11	73.3%
7. Did the review authors provide a list of excluded studies and justify the exclusions?∗	3	20.0%
8. Did the review authors describe the included studies in adequate detail?	15	100.0%
9. Did the review authors use a satisfactory technique for assessing the risk of bias (RoB) in individual studies that were included in the review?	12	80.0%
10. Did the review authors report on the sources of funding for the studies included in the review?∗	3	20.0%
11∗. If meta-analysis was performed, did the review authors use appropriate methods for statistical combination of results?	15	100.0%
12. If meta-analysis was performed, did the review authors assess the potential impact of RoB in individual studies on the results of the meta-analysis or other evidence synthesis?	8	53.3%
13. Did the review authors account for risk RoB in individual studies when interpreting/discussing the results of the review?∗	10	66.7%
14. Did the review authors provide a satisfactory explanation for, and discussion of, any heterogeneity observed in the results of the review?	15	100.0%
15. If they performed quantitative synthesis, did the review authors carry out an adequate investigation of publication bias (small study bias) and discuss its likely impact on the results of the review?∗	9	60.0%
16. Did the review authors report any potential sources of conflict of interest, including any funding they received for conducting the review?	14	93.3%

*PICO*, Population, Intervention, Comparison, and Outcome.

∗ Indicates a critical domain in the AMSTAR 2 assessment of reviewed studies.

### Frequency of spin and analysis

**Figure 3:**
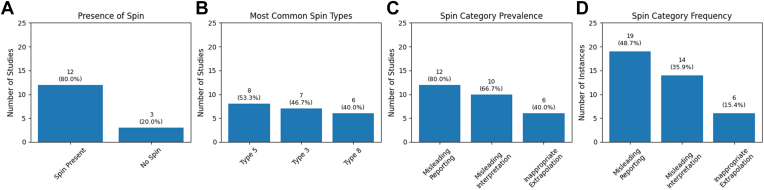
Analysis of spin bias among the reviewed studies. (**A**) Presence of spin in the included studies. (**B**) Evaluation of the most common types of spin appearing across studies. (**C**) Prevalence of spin bias categories across the included studies. (**D**) Frequency of spin bias categories represented as a percentage of instances or occurrences.

Only 3 studies (20.0%) did not contain spin, while spin was determined to have been present in the abstracts of 12 out of 15 studies (80.0%) (Fig. 3*A*). Spin types 2, 6, 7, 13, 14, and 15 were not observed in any of the studies included. The median number of spin types identified per study was 3 (range: 0-5, mean = 2.60 ± 1.55). The 3 most common types of spin were types 5 (present in 8 out of 15 studies, 53.3%), 3 (present in 7/15 studies, 46.7%), and 8 (present in 6/15, 40.0%) (Table IV; Fig. 3*B*). The category of spin that was most prevalent was misleading reporting (which included spin types 3, 5, 6, 10, 11, 13, and 14); this category was present in 12 out of the 15 studies (80.0%) (Fig. 3*C*). Misleading reporting also had the highest frequency, with a total of 19 out of 39 instances across the 15 studies (48.7%) (Fig. 3*D*). Ten of 15 (66.7%) studies contained spin within the category of misleading interpretation (spin types 1, 2, 4, 9, and 12). The frequency of misleading interpretation was lower than that of misleading reporting, with a total of 14/39 instances across the 15 studies (35.9%). Finally, 6/15 (40.0%) studies contained spin within the category of inappropriate extrapolation (spin types 7, 8, and 15), which was found at a frequency of 6 out of 39 instances across the 15 studies (15.4%).

**Table IV:** Frequency of spin type and category.

Category	Type	Description	Abstracts
Misleading interpretation	1	The conclusion formulates recommendations for clinical practice not supported by the findings	2 (13.3%)
	2	The title claims or suggests a beneficial effect of the experimental intervention not supported by the findings	0 (0%)
	4	The conclusion claims safety based on nonstatistically significant results with a wide confidence interval	4 (26.7%)
	9	The conclusion claims the beneficial effect of the experimental treatment despite reporting bias	3 (20.0%)
	12	The conclusion claims equivalence or comparable effectiveness for nonstatistically significant results with a wide confidence interval	5 (33.3%)
Misleading reporting	3	Selective reporting of or overemphasis on efficacy outcomes or analysis favoring the beneficial effect of the experimental intervention	7 (46.7%)
	5	The conclusion claims the beneficial effect of the experimental treatment despite a high risk of bias in primary studies	8 (53.3%)
	6	Selective reporting of or overemphasis on harm outcomes or analysis favoring the safety of the experimental intervention	0 (0%)
	10	Authors hide or do not present any conflict of interest	1 (6.7%)
	11	The conclusion focuses selectively on statistically significant efficacy outcome	3 (20.0%)
	13	Failure to specify the direction of the effect when it favors the control intervention	0 (0%)
	14	Failure to report a wide confidence interval of estimates	0 (0%)
Inappropriate extrapolation	7	The conclusion extrapolates the review findings to a different intervention (eg, claiming efficacy of one specific intervention although the review covered a class of several interventions)	0 (0%)
	8	The conclusion extrapolates the review's findings from a surrogate marker or a specific outcome to the global improvement of the disease	6 (40.0%)
	15	The conclusion extrapolates the review's findings to a different population or setting	0 (0%)

### Statistical tests of association

The associations between Level of Evidence and the presence of spin (*P* = .0967) or the number of spin types present (*P* = .1070) were not statistically significant (Table V). However, there was a statistically significant association between the year of publication and both the overall presence of spin (*P* = .0078, r = −0.657, 95% CI: [-0.878, −0.380]) and the number of spin types present (*P* = .0168, r = −0.605, 95% CI: [-0.858, −0.151]), where the occurrence of spin and the number of spin types present decreases with newer studies.

**Table V:** Statistical analysis with corresponding *P* values for each test performed.

Analysis	Statistical test	*P* value	Significance
Level of evidence vs. presence of spin	Fisher exact test	.0967	Not significant
Level of evidence vs. number of spin types	Kruskal-Wallis test	.107	Not significant
Year of publication vs. presence of spin	Point-biserial correlation	**.0078**	Significant
Year of publication vs. number of spin types	Spearman correlation	**.0168**	Significant
AMSTAR 2 confidence rating vs. presence of spin	Ordinal logistic regression	.9288	Not significant
AMSTAR 2 confidence rating vs. number of spin types	Mann-Whitney U test	.9033	Not significant
AMSTAR 2 numerical rating vs. presence of spin	Mann-Whitney U test	.3835	Not significant
AMSTAR 2 numerical rating vs. number of spin types	Spearman correlation	.197	Not significant
Impact factor vs. presence of spin	Logistic regression	.6261	Not significant
Impact factor vs. number of spin types	Spearman correlation	.4079	Not significant

Statistically significant *P* values are bolded.

The AMSTAR 2 assessment identifies critical flaws in systematic reviews and meta-analyses by assigning studies critically low, low, moderate, and high confidence ratings. This study found that the AMSTAR 2 confidence rating was not significantly associated with the presence of spin (*P* = .9288). The AMSTAR 2 confidence rating was also not significantly associated with the number of spin types present (*P* = .9033). There was no statistically significant association between the numerical AMSTAR 2 rating and the overall presence of spin (*P* = .3835). The average numerical AMSTAR 2 score for studies with no spin was 13.0 ± 2.65 (range: 11-16), while the average for studies with spin was 11.1 ± 3.34 (range: 6-16). As the number of spin types present increased, the AMSTAR 2 rating tended to decrease. However, this finding was also not statistically significant (*P* = .1970, r = −0.353).

There was no statistically significant association between the journal impact factor and the presence of spin. The average impact factor of the studies that had spin present was 2.46 ± 1.56, while the average impact factor of the studies that did not have spin present was 2.00 ± 0.92 (*P* = .6261). As the number of spin types present increased, the impact factor of the journal the study was published in tended to decrease. However, this finding was not statistically significant (*P* = .4079, r = −0.2308).

## Discussion

This study demonstrates a high prevalence of spin in systematic reviews and meta-analyses evaluating the use of TXA in shoulder arthroplasty. Nearly 80% of abstracts analyzed contained at least one form of spin, with spin types 3 (“selective reporting of or overemphasis on positive efficacy outcomes or favorable secondary outcomes”), 5 (“the conclusion claims benefit despite a high risk of bias in primary studies”), and 8 (“inappropriate extrapolation of findings”) being the most frequently identified. Spin type 5 was determined to be present if the study claimed the benefit of TXA without mentioning an elevated bias from their risk of bias assessment. Studies that exhibited spin type 3 typically concluded that their statistically significant findings should be implemented as an intervention. For example, Hartland et al may have overemphasized the reduced blood loss from using TXA during shoulder surgery, as only one of the included studies had a low risk of bias without any concerns, albeit further called for the need for larger randomized control trials with low risk of bias in future studies.^8^ Some studies tended to extrapolate their findings to a global improvement of disease leading to spin type 8. Kuo et al concluded that TXA for TSA does not increase complications or thromboembolic events, yet their statistical analysis reported no significant difference between patients who received TXA and those who did not.^12^ Misleading reporting and interpretation were particularly prominent, observed in 80.0% and 66.7% of studies, respectively. These findings align with prior analyses in orthopedic literature, where spin has been identified across various surgical topics. Gulbrandsen et al identified spin in 52.8% of abstracts of systematic reviews related to midshaft clavicle fractures, with spin type 3 being the most common.^7^ Similarly, Thompson et al identified spin in 100% of abstracts assessing systematic reviews on ulnar collateral ligament reconstruction, with spin type 5 accounting for 36.8% of cases.^20^ Kumaran et al expanded upon this by identifying spin type 9 as the most prevalent in studies on lateral extra-articular tenodesis and anterolateral ligament reconstruction.^11^ Collectively, these studies highlight a broader trend in orthopedic surgical literature toward overstating positive findings, potentially influencing clinical decision-making based on flawed or biased interpretations of the original evidence.

In addition to the prevalence of spin, the methodological rigor of the included studies was suboptimal. The AMSTAR 2 evaluation revealed that 60.0% of systematic reviews were classified as critically low confidence, with only 26.7% achieving a high confidence rating. This reflects significant methodological deficiencies consistent with prior orthopedic literature. Although the AMSTAR 2 score was quantified and compared for the studies that had spin bias with studies which had no spin bias, the failure of studies to meet the critical domain criteria is more suitable to explain a given study's overall confidence. Only 4 studies (26.7%) had their protocols registered prior to beginning their review or meta-analysis, and 3 studies (20.0%) justified their exclusions with a list of the excluded studies for full transparency. This suggests many of the studies that received a critically low confidence were in part due to critical domain failures. There is no evidence to suggest that spin bias is associated with AMSTAR 2 confidence ratings, and this study supported this notion as the statistical analysis showed no statistically significant association between the two. Shea et al, in their foundational work introducing AMSTAR 2, emphasized the importance of comprehensive methodological evaluation in systematic reviews.^17^ Despite improvements in reporting tools such as PRISMA,^13^ adherence to these guidelines remains inconsistent, particularly in older studies or those published prior to the widespread endorsement of PRISMA standards by journals.

Interestingly, while spin has often been shown to occur regardless of journal impact factor, prior research suggests that lower-quality studies may be preferentially published in lower-impact journals, which may also correlate with weaker editorial oversight regarding spin detection.^11^^,^^20^ Our statistical analysis for this study of the selected intervention found no relationship between spin presence and journal impact factor. Nevertheless, the presence of spin even in higher-impact journals underscores the necessity for ongoing critical appraisal by both peer reviewers and readers. Our analysis did reveal a statistically significant temporal trend, such that both the prevalence of spin and the number of types of spin in given studies are decreased in more recent publications compared to earlier studies. However, this does not indicate that spin bias is decreasing in the literature. Additionally, this finding may be confounded by variation in editorial policies across journals, the adoption of PRISMA standards, and increased methodological scrutiny from journal reviewers and editors over time.

The routine use of TXA in shoulder arthroplasty continues to increase, particularly as surgeons aim to reduce perioperative blood loss and transfusion rates.^21^^,^^25^ While multiple systematic reviews and meta-analyses support TXA's clinical utility in shoulder arthroplasty,^21^^,^^25^ our findings suggest that clinicians should approach conclusions drawn from these studies with caution. Overstated abstracts may create undue confidence in the efficacy or safety of TXA, potentially influencing surgical decision-making prematurely or inappropriately. Boutron et al demonstrated that misleading abstracts could influence clinicians' interpretations of surgical literature, reinforcing the importance of accuracy and transparency in reporting.^2^

Future efforts should focus on improving the methodological rigor of systematic reviews, ensuring adherence to validated reporting guidelines such as PRISMA, and increasing awareness of reporting bias among authors, reviewers, and journal editors.^11^^,^^14^^,^^20^ By addressing these shortcomings, the orthopedic community can work toward more reliable syntheses of evidence regarding TXA use in shoulder arthroplasty, ultimately supporting better-informed clinical decisions.

### Limitations

There are several limitations to the findings of this study, including some introduced by the methodology. Because studies not published in the English language were excluded, there was potential for language bias, as excluding such articles may lead to an incomplete assessment of the current evidence. For instance, the process of identifying spin is subjective, despite measures taken to mitigate bias, such as the simultaneous collection process by 3 reviewers in this study. Another methodological limitation to the study was that there was no formal calculation for inter-rater reliability statistics for the authors' ratings of spin bias and AMSTAR 2. Instead, this study was reliant on a structured collaboration process with consensus-based decisions, which may introduce subjectivity. This study may have also been somewhat limited by the level of evidence of the included studies: while 33.3% of the studies had a Level of Evidence of I, over half (53.3%) presented only a Level of Evidence of III. Furthermore, intrinsic limits to certain study characteristics exist, for example, that many studies were published prior to the release of PRISMA guidelines. It is unclear exactly when journals started endorsing the PRISMA criteria. Additionally, the AMSTAR 2 instrument employed for evaluation was created and released in 2017; using it to evaluate systematic reviews that were published earlier in 2017 would have led to a lower quality of conclusions. Finally, this study risks the presence of false positives in its statistically significant findings for publication year and spin presence as well as the number of spin types present, as a sample size of 15 studies is very low. Similarly, the findings that newer studies contained fewer spin types with r = −0.657 should be considered regarding the overall small sample size. Therefore, these significant findings must be interpreted as a hypothesis until a larger sample size may be created through performing more studies.

## Conclusion

This study highlights a concerning prevalence of spin within systematic reviews and meta-analyses assessing the use of TXA in shoulder arthroplasty. Despite growing clinical interest in TXA for reducing perioperative blood loss, our findings reveal that a large majority of these reviews exhibited misleading reporting (80.0%) or misleading interpretation (66.7%), representing a frequent and widespread overstatement of evidence supporting the efficacy of TXA. While the use of TXA remains safe and widely supported for shoulder arthroplasty, the writing of systematic reviews and meta-analyses should be performed carefully to avoid misinterpretation of results due to underlying biases. Furthermore, the overall methodological quality of these studies, as assessed by AMSTAR 2, was frequently suboptimal (median score of 11), with 80.0% of included studies receiving a critically low AMSTAR 2 confidence rating. These shortcomings underscore the importance of critical appraisal when interpreting systematic reviews on TXA in shoulder arthroplasty. Additionally, this study of 15 systematic reviews and/or meta-analyses on the topic of TXA use in shoulder arthroplasty raises the question if the literature is becoming redundant and diluted, as they each essentially are asking the same questions. Future research should emphasize methodological rigor, strict adherence to established reporting guidelines such as PRISMA, protocol registration prior to conducting the study, and vigilance in avoiding spin through transparent reporting to ensure that clinicians and stakeholders are guided by the most reliable and unbiased evidence available.

## Disclaimers:

Funding: No funding was disclosed by the authors.

Conflicts of interest: The authors, their immediate families, and any research foundations with which they are affiliated have not received any financial payments or other benefits from any commercial entity related to the subject of this article.

## Supplementary Data

Data Extraction
